# Optimizing CNC turning of AISI D3 tool steel using Al₂O₃/graphene nanofluid and machine learning algorithms

**DOI:** 10.1016/j.heliyon.2024.e40969

**Published:** 2024-12-05

**Authors:** Leta Daba Gemechu, Dame Alemayehu Efa, Robsan Abebe

**Affiliations:** School of Mechanical Engineering, Institute of Technology, Wallaga University, P.O. Box 395, Nekemte, Ethiopia

**Keywords:** AISI D3 tool steel, CNC turning, Hybrid nanofluid, Machine learning, Optimization

## Abstract

Turning AISI (American Iron and Steel Institute) D3 tool steel can be challenging due to a lack of optimal process parameters and proper coolant application to achieve high surface quality and temperature control. Machine learning helps in predicting the optimal parameters, whereas nanofluids enhance cooling efficiency while preserving both the tool and the workpiece. This work intends to utilize advanced machine learning approaches to optimize process parameters with the application of hybrid nanofluids (Al_2_O_3_/graphene) during the CNC turning of AISI D3. The Response Surface Methodology (RSM), Back Propagation (BP) neural networks, and Genetic Algorithms (GA) will be utilized to model and predict optimal turning parameters to enhance surface quality and manage tool tip temperature. The experiments ranged the cutting speed, nanofluid concentration, depth of cut, and feed rate from 150 to 180 m/min, 0.3 to 0.9 wt%, 0.5–0.9 mm, and 0.03–0.07 mm/rev. RSM and ANN analyses showed that cutting speed and feed rate had a significant effect on surface quality, contributing 11.5 % and 10.5 %, respectively, whereas the nanofluid affected tool tip temperature by 42.5 %. The GA determined that the optimal cutting speed became 150 m/min, the feed rate was 0.05 mm/rev, the cutting depth was 0.6 mm, and the nanofluid concentration was 0.8 %. At temperatures ranging from 23.01 °C to 28.41 °C, these conditions produced a desirable surface roughness of 0.16–0.45 μm. The findings emphasize the benefits of utilizing Al_2_O_3_/graphene nanofluid and machine learning algorithms in CNC turning to improve surface roughness and control temperature.

## 1 Introduction

A nanofluid is a fluid that contains suspended nanoparticles with diameters smaller than 100 nm, disseminated in a base fluid like water, oil, or ethylene glycol. Its efficient adoption in the machining process demands careful thought of aspects such as nanoparticle concentration, dispersion stability and compatibility with cutting equipment to ensure maximum performance and safety [1,2].

**Table:** Nomenclature

ϕ (wt %)	weight percentage of the nanoparticles in the nanofluid (wt. %)
m	mass of the nanoparticle for each nanofluid (kg)
ρAl_2_O_3_	density of the aluminum oxide (kg/ $m^{3}$)
ρ_bf_	density of the base fluid (kg/ $m^{3}$)
v	cutting speed (m/ min)
β_0_	constant
f	feed rate (mm/rev)
d	depth of cut (mm)
T	temperature (°C)
Ra	surface roughness (μm)
β_1_, β_2_, β_3_	linear term coefficient

Turning is commonly utilized in the machining industry, to efficiently remove material and yield superior surface quality [3]. However, to achieve high surface quality and effective temperature control in CNC turning, machining parameters should be precisely optimized [4]. Industries prioritize cost-effective manufacturing by selecting turning process parameters like spindle velocity, feed rate, and depth of cut to ensure consistent surface texture [5]. CNC machining enhances the surface finish and dimensional accuracy, utilizing tough and hard tools [6]. Specifically, AISI D3 Steel, with significant chromium, nickel, molybdenum, and carbon, has excellent wear resistance; though achieving optimal results is also dependent on other factors [7].

Conventional cutting fluids lead to additional costs, reduced productivity, adverse effects on operator health, and increased environmental concerns [8]. Minimum Quantity Lubrication (MQL) provides a tiny mist of lubricant directly to the cutting zone, considerably reducing fluid consumption, machining costs and environmental effects [9,10]. Additionally, it enhances machining performance and increases tool life by minimizing friction and heat. Roy et al. [11] found that MQL and cryogenic cooling improve machinability and tool lifespan for machining difficult-to-cut metals. Padha et al. [12] found that MQL greatly improved the turning of Nitronic 60 steel with SiAlON ceramic tools, increasing tool life to 81 min while decreasing cutting forces, temperatures, and tool wear when compared to flooded, compressed-air, and dry conditions. However, nanofluid-assisted MQL outperforms dry and conventional MQL machining by improving lubrication and cooling utilizing nanoparticles [13,14], resulting in a better surface finish [15] and reduced cutting forces, tool wear [16] and cutting temperature [[17], [18], [19]]. This results in a more efficient and environmentally friendly machining process. Gupta & Korkmaz [20] provided a conceptual framework for assessing the MQL and hBN-enriched Nano-MQL to employ hBN-enriched nanofluids reduced tool wear values to 26.9 % compared to dry machining. Dash et al. [21] evaluated the hard turning of AISI D3 steel using a coated carbide tool and nanofluid MQL. They observed that nanofluid-based MQL increased tool life by 30.8 % and decreased surface roughness by 22.6 % compared to conventional MQL.

Increasing nanoparticle concentration significantly reduces tool wear, surface roughness, power consumption, and temperature, leading to improved processing efficiency, extended tool life, and enhanced thermal conductivity [22,23]. Kanti et al. [24] found that graphene Ionanofluid (INF) at a 0.5 wt% concentration performs well in thermal applications, with enhanced density, higher thermal conductivity, and lower viscosity. In their study, Kanti et al. [25] observed that graphene Ionanofluid enhanced thermal conductivity by 32.9 % at 60 °C and viscosity by 76.3 % at 30 °C by gene expression programming. Ross et al. [26] found that employing multi-walled carbon nanotubes in vegetable oil as a cutting fluid for machining Monel 400 reduced cutting temperature by around 65 % compared to dry machining, improving surface roughness and reducing tool wear. Gupta et al. [27] established an approach to assess the sustainability of machining Bohler K490 steel with hBN-enriched nanofluids, which reduced tool wear by 26.9 %, surface roughness by 17 %, and power consumption while enhancing overall sustainability. Mahapatra et al. [28] observed that employing an S3P-AlTiSiN coated tool with MWCNT nanofluid improves tool life to 42 min while reducing the cost to Rs. 153.52 per part, with nose radius affecting roughness and cutting speed affecting vibration. Roy et al. [29] reported that nanofluid-MQL machining of AISI 4140 steel with a coated carbide tool reduces power consumption, cutting force, temperature, and flank wear while providing superior surface quality and sustainability than dry and flooded cutting.

The integration of RSM, GA, and ANN enhances turning operations by efficiently optimizing parameters and accurately predicting outcomes, leading to improved performance and productivity [30] optimized turning process parameters of AISI 1040 steel in a dry environment using GA, which is valued at 1.478 % flank wear and 1.146 % f surface roughness [31]. The RSM, which is utilized in Design-Expert software for mathematical modeling and optimization, was used to AISI D3 steel to achieve the optimum cutting speed (140 m/min), feed rate (0.01 mm/rev), and depth of cut (1 mm) throughout the machining process [32]. Adopting different lubrication conditions guided by RSM and a hybrid deep recurrent neural network-black widow optimizer (DRNN-BWO) prediction model enhanced the machinability of SS304 alloy steel during turning operations [33]. Korkmaz et al. [34] developed machine learning models to identify wear and friction in stainless steel 316L, with the J48 model achieving near-perfect accuracy, despite significant wear occurring under high load when MQL was insufficient. Korkmaz et al. [35] revealed that doubling the cutting speed increased tool wear by 44.4 % in AA7075 aluminum turning, with image processing exhibiting a 3.5 % divergence and the Multilayer Perception (MLP) model varying by 5 % from the linear regression (LR) model. Gupta et al. [36] employed deep learning to predict the tribological properties of additively manufactured 316 stainless steel, revealing that it had a 58 % lower wear rate than casted material, with the Convolutional Neural Network (CNN) and Attention-based CNN models providing the highest accuracy due to improved microstructure. Gupta et al. [37] used a machine-learning model to investigate the tribological attributes of 316L steel versus 100Cr6 alloy. They showed that combining MQL with cryogenic cooling diminished friction forces by more than ten times for sliding distances greater than 30 m and loads less than 25 N, significantly improving performance. Korkmaz et al. [38] observed that MQL reduces flank wear by 5 %–25 % when machining Bohler steel, with machine learning models identifying the optimal cutting conditions. Yurtkuran et al. [39] utilized machine learning to investigate power consumption during the milling of PH13-8Mo stainless steel and discovered that higher feed speeds increased power consumption by 3.14 %, whereas different lubrication conditions reduced power consumption compared to dry cutting. Chauhan et al. [40] employed crayfish and arithmetic optimization to predict the friction behavior of Ti-6Al-4V alloy, and the Support Vector Machine (SVM) model achieved 95.85 % accuracy in 26.85 s. Kanti et al. [41] observed that thermal conductivity (TC) increased with temperature and concentration, with the hybrid nanofluid outperforming fly ash while ANN and Multi-gene Genetic Programming (MGGP) models accurately predicted TC. Kanti et al. [42] reported that the hybrid nanofluid had higher viscosity than the fly ash nanofluid and that the MGGP model outperformed ANN and linear regression in predicting viscosity, with high correlation coefficients and low error values. Kumar et al. [43] reported that red mud nanofluids enhanced solar collector efficiency by improving thermal conductivity by 36.9 % at 0.75 vol% concentration, utilizing machine learning to make precise property predictions.

Generally, the metal-cutting industry is shifting the harmful traditional cutting fluids to eco-friendly Nano fluids due to environmental factors [44]. While there has been extensive research on these environmentally friendly fluids, the majority of the studies have focused on process parameters for various materials in turning processes. However, to the best of the authors' understanding, there is a significant research gap in using hybrid nanofluids (Al_2_O_3_/graphene) and machine learning algorithms to optimize the CNC turning process of AISI D3 tool steel. This study investigates the use of Al₂O₃/graphene nanofluids in CNC turning, focusing on machine learning algorithms to enhance surface roughness and control cutting temperature.

## 2 Materials and methods

### 2.1 Materials

Selecting the right materials for CNC turning, especially when using hybrid nanofluids, becomes essential for increasing efficiency, quality, and cost-effectiveness [45]. Mechanical properties such as strength and hardness are important aspects of determining cutting conditions and surface quality [46]. AISI D3 tool steel contains high carbon and chromium (as seen in Table 1) chosen due to its exceptional wear resistance, hardness, and dimensional stability at high temperatures [7,17]. The carbide insert is utilized during CNC turning operation due to its durability, wear resistance, and thermal stability [[47], [48], [49]]. The materials and equipment used in this study are listed in Table 2.

**Table 1:** Chemical composition of AISI D3 tool steel [7].

Elements	C	Mn	Si	Ni	Cr	S	P	Vn	Ferrous
Amount (wt. %)	1.973	0.354	0.32	0.265	11.463	0.009	0.016	0.047	Remainder

**Table 2:** Experimental setup and equipment details

Materials	Details
Workpiece Material	AISI D3 tool steel: Ø40 mm, L110 mm, 45 HRC
CNC Lathe Machine	DMTG CNC lathe, model CKE 6150: 3-phase, 380V, 50 Hz.
Surface Roughness Tester	VOGEL, Model 65711
Cutting Tool Material	**Carbide Inserts:** CNMG431-MA (CNMG120404)
	**Dimensions:** 12 × 5 mm (0.472 × 0.196 inches)
Tool holder	MCGNR-M12
Tool Tip Temperature Measurement	ATAL infrared Type K thermometer: Emissivity set to 0.28 [50]
Nanofluids	Al₂O₃/graphene
Homogenizer & Stabilizer for hybrid nanofluids	Ultrasonicator

### 2.2 Methodology

#### 2.2.1 Preparation of nanofluids

**Fig. 1:**
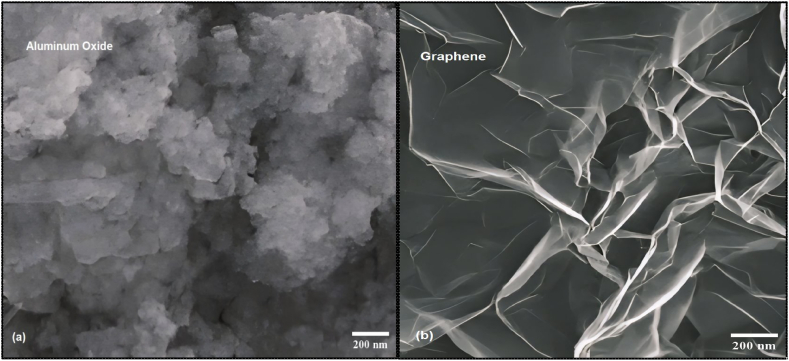
Scanning Electron Microscope (SEM) images of nanomaterials (a) Al_2_O_3_ (b) Graphene.

The microstructure of aluminum oxide (Al₂O₃) nanoparticles and graphene nanoplatelets is essential for applications in CNC turning operations. These nanoparticles have spherical forms and range in size from 15 to 45 nm [51]. In contrast, graphene nanoplatelets have a flat, layered structure that maintains a thin profile while varying in size [52]. When graphene nanoplatelets are dispersed in aluminum composites, their mechanical properties improve due to the flat-like structure [53]. Both materials are employed to improve the physical properties and tribological performance of materials, resulting in higher wear resistance and strength in CNC turning applications [52,53]. Fig. 1 (a) and (b) present the SEM images of the aluminum oxide (Al₂O₃) and graphene nanomaterials analyzed in this study.

Nanofluid has been obtained by dispersing nanoparticles into a base fluid for proper dispersion. Equation (1) determined the ideal ratio for creating hybrid nanofluids to deionize water and glycerol solvents [54].

The Al₂O₃/graphene nanofluids were purchased from Adnano Technologies Pvt. Ltd., India, and were prepared using a two-step process.

(1) $$\phi \left(\;\text{wt}.\;\%\right)=\frac{\left(\frac{m}{\rho \;}\right){\text{Al}}_{2}O_{3}+\left(\frac{m}{\rho }\right)\text{graphene}}{\left(\frac{m}{\rho }\right){\text{Al}}_{2}O_{3}+\left(\frac{m}{\rho }\right)\text{graphene}+\left(\frac{m_{bf}}{\rho_{bf}}\right)\;}$$

**Fig. 2:**
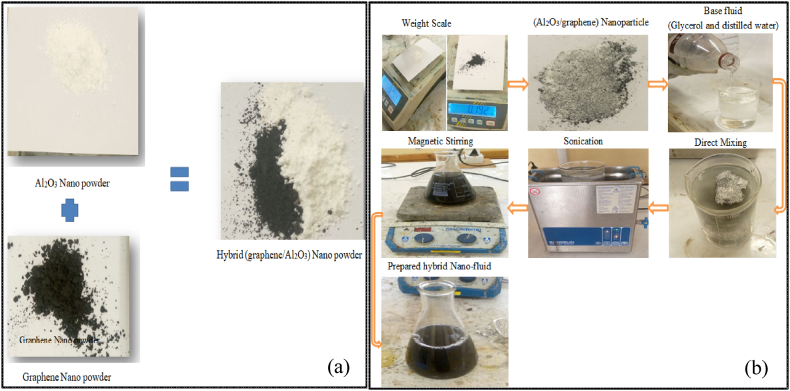
Preparation of hybrid graphene/Al_2_O_3_ nanopowder (a) nanoparticle mixing and (b) nanofluid preparation process.

Based on the physical and chemical properties of the nanoparticles indicated in Table 3, Fig. 2 (a) and (b) illustrate the preparation of 3 L of glycerol and deionized water in a 25:75 ratio. The nanofluid was generated at three distinct concentrations: 0.3 %, 0.08 g of graphene and 1.15 g of Al_2_O_3_; 0.6 %, 0.15 g of graphene and 2.25 g of Al_2_O_3_; and 0.9 %, 0.225 g of graphene and 3.375 g of Al_2_O_3_ in addition, graphene was mixed into the nanofluid at a 75:25 ratio. Several methods are used in this study to ensure nanofluid stability, including magnetic stirring and ultrasonication [[56], [57], [58]], keeping nanoparticle concentration below 2 % to reduce the possibility of agglomeration [57] and conducting regular qualitative inspections to monitor dispersion stability [59].

**Table 3:** Physical properties of nanoparticles [55].

Material Properties	Values (Al_2_O_3_)	Values (graphene)
Bulk density	0.5 g/cm^3^	0.166 g/cm^3^
Form of particle	Powder	Powder
Average Particle Size	20–50 nm	<10 nm
Purity	99 %	99 %
Nanoparticle Color	White	Greyish Black

#### 2.2.2 Experimental design

Experimental design is a crucial aspect of research methodology, ensuring the reliability and validity of results by structuring experiments to optimize data quality and interpretability. Sample size estimation is an important aspect, as is using tools like the ExpDesign software to determine optimal sizes and choose appropriate designs, such as randomized blocks and factorial designs [60]. Table 4 illustrates the input process parameters used in this experiment, as well as their levels and ranges. These process parameter ranges were selected based on manufacturer recommendations [61], optimization goals [62], experimental validation [63], material hardness [64] and machining relevance [65].

**Table 4:** Process variables and their levels

Cutting Variables	Levels		
	−α	0	+α
Cutting speed	150	165	180
Depth of cut	0.5	0.7	0.9
Feed rate	0.03	0.05	0.07
Concentration of nanoparticle	0.3	0.6	0.9

#### 2.2.3 Optimization methodology

**Fig. 3:**
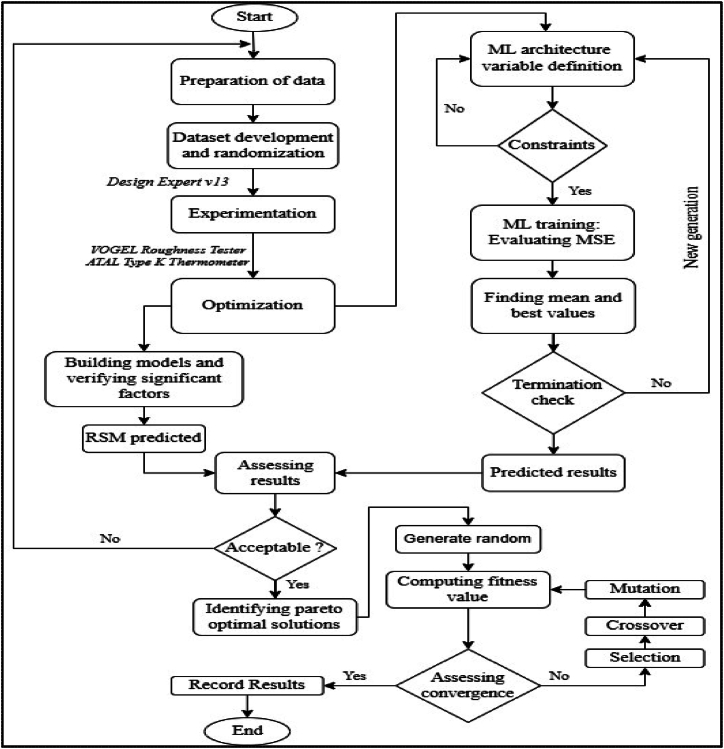
Flowchart of the optimization process for tool tip temperature and surface roughness in CNC turning.

The optimization methodology used in this work combines experimental data, statistical analysis, machine learning, and genetic algorithms to optimize machining conditions. Fig. 3 presents a flowchart detailing the steps required for optimizing tool tip temperature and surface roughness in CNC turning, considering various parameters such as cutting speed, feed rate, depth of cut, and nanofluid concentration.

##### 2.2.3.1 Response surface methodology (RSM)

RSM is a statistical tool that optimizes processes by simulating the interactions between multiple input variables and responses. To systematically investigate how various input factors influence outputs, RSM employs techniques such as Central Composite Design (CCD) and Box-Behnken Design (BBD) [30]. The CCD combines factorial and axial points to enhance precision in parameter estimates and improve optimization [66]. These designs help to identify significant variables influencing responses, allowing for more efficient experimentation. RSM typically employs polynomial regression models to approximate the response surface, which helps to understand complex interactions between variables [67]. Equation (2) explains the relationship between a quadratic polynomial's response variable (*y*) and process variable (*x*) [68].

(2) $$Y=\beta_{0}+\beta_{1}x_{1}{+\beta }_{2}x_{2}{+\beta }_{3}x_{3}+\beta_{11}x_{12}+\beta_{22}x_{22}{+\beta }_{12}x_{1}$$

Analysis of variance (ANOVA) is commonly utilized to determine the significance of factors and interactions. Results are analyzed using graphical representations such as contour plots, which visually depict the optimal conditions for desired outcomes [69]. The methodology has been successfully used in numerous fields, including nanomaterial optimization and environmental remediation [[66], [67], [68], [69], [70]]. Therefore, this study utilized a CCD with a face-centered configuration, incorporating three levels per factor and six center points to enhance prediction accuracy.

##### 2.2.3.2 Artificial neural network (ANN)

In this study, MATLAB R2022b was utilized for the development and implementation of the ANN. The Levenberg-Marquardt (LM) algorithm was used for training [71,72], with specific parameters set for weight assignment and normalization, such as LEARNGDM (adaptation function), MSE (performance function), and TANSIG (transfer function) [30]. This technique optimizes parameters dynamically during the training process, resulting in greater convergence speed and higher accuracy.

**Fig. 4:**
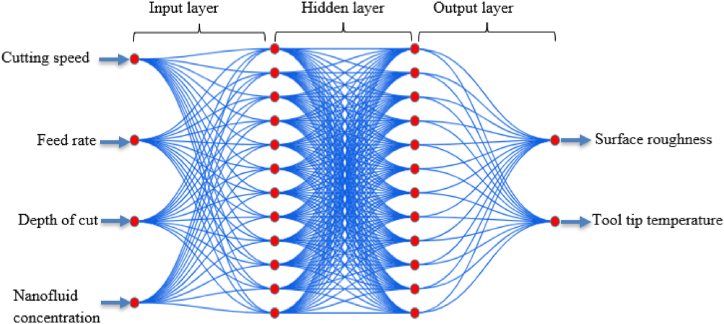
Structure of a multi-layer perceptron.

The LM approach achieves local quadratic convergence under specific conditions, making it effective for determining parameters in complex problems [73]. In the context of hybrid nanofluids, the LM backpropagation (BP) approach effectively maps complex physical constraints [74]. The weight initialization matrix is [150 180; 0.03 0.07; 0.5 0.9; 0.3 0.9]. Model training involves specifying the number of epochs, with a maximum failure limit of 1000. The quantity of neurons in the output layer depends on the complexity of the task [75]. Fig. 4 illustrates the supervised learning models of a BP neural network with one input layer comprising four input parameters, two hidden layers each containing twelve units and one output layer with two output units.

##### 2.2.3.3 Genetic algorithm (GA)

GA is becoming more commonly utilized for multi-objective parametric optimization in machining [30,76]. They excel in navigating complex solution spaces to identify the most optimal combinations of variables, which are essential for achieving desired results like enhanced surface finishes and longer tool life. GAs can optimize numerous different objectives at the same time, such as decreasing surface roughness while determining temperature rise during turning operations [77]. GAs can be used to identify Pareto optimum solutions, which are the best trade-offs between multiple goals [78]. The dataset is critical for guiding iterative improvements in a GA, allowing it to analyze various regions of the input space using modeled relationships. Several key parameters are set for the GA during this process, including a population size of 55 individuals, a crossover rate of 0.8, a constant-dependent mutation function, an intermediate crossover function, and a tournament selection method. The algorithm starts with a randomly generated set of potential solutions. Each individual in this population gets assessed using a fitness function to determine their quality. Individuals are chosen based on these evaluations to produce offspring for the next generation. The chosen individuals then undergo crossover and mutation, resulting in a new generation of solutions. This iterative process continues for a set number of generations or until the solutions reach a satisfactory level of fitness. The parameter ranges and multi-objective parametric optimization can be described by Equation (3). Additionally, the genetic algorithms efficiently explore the solution space for optimal parameter selection while ANN validates the model by learning patterns, predicting responses, and confirming RSM findings [68].

(3) $$\left\{ \begin{array}{c} 150m/\min\leq V\leq 180m/\min\\ 0.03\text{mm}/\text{rev}\leq V\leq 0.07\text{mm}/\text{rev}\\ 0.5mm\leq d\leq 0.9mm\\ 0.3\%\leq \phi \leq 0.9\% \end{array}\right.$$

## 3 Results and discussion

### 3.1 Results of the study

**Fig. 5:**
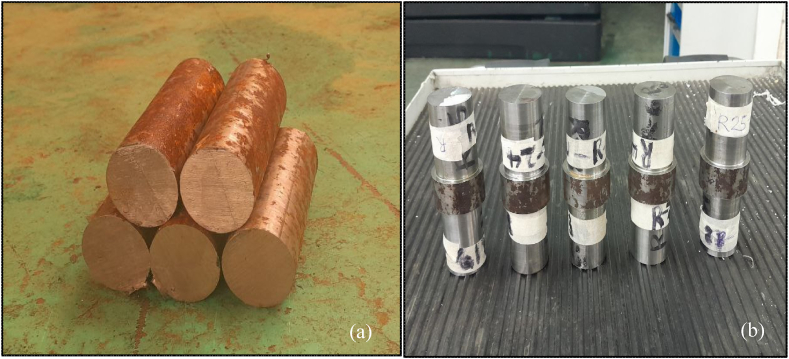
Workpiece (a) before and (b) after turning.

The study focuses on the advantages of incorporating a hybrid Al_2_O_3_/graphene nanofluid to increase machining performance. This nanofluid was used during the turning of AISI D3 steel on a CNC lathe with a carbide insert cutting tool. Fig. 5 (a) and (b) represent the workpiece before and after turning.

**Fig. 6:**
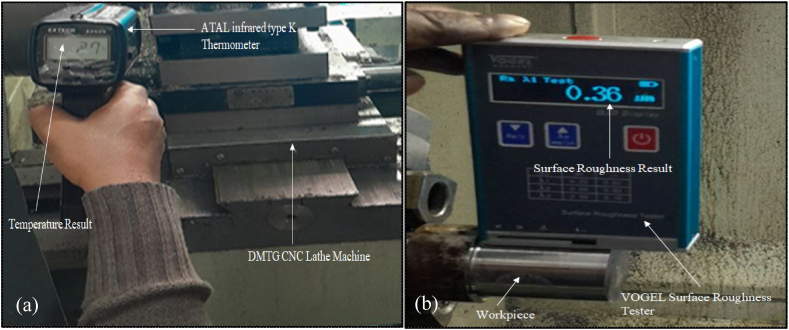
Measuring of (a) temperature and (b) surface roughness.

**Fig. 7:**
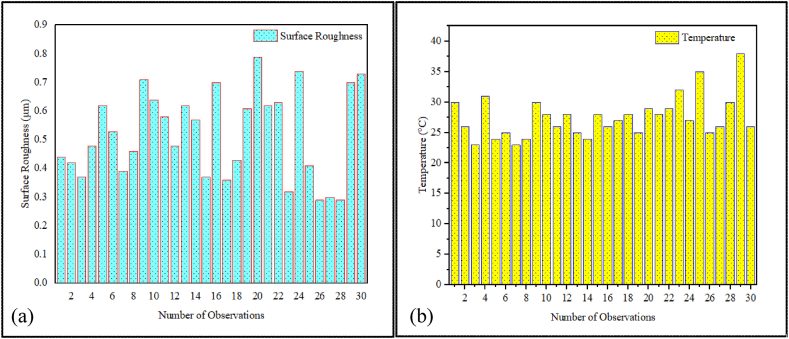
The visual representations of (a) Temperature and (b) Surface Roughness results for the 30 observations, each with different input variables.

Fig. 6 (a) and (b) illustrate temperature and surface roughness results along with sample images, whereas Fig. 7 (a) and (b) show the graphical representations for 30 observations. Table 5 presents the results of experiments conducted during the turning of AISI D3 tool steel. During the experiment, the cutting speed, feed rate, nanoparticle concentration and depth of cut were adjusted correspondingly to investigate surface roughness and temperature across 30 different specimens.

**Table 5:** CNC lathe turning operation of experimental result

Run	v	f	d	ϕ	Ra (μm)				T (°C)
					3 (replicate tests of Ra)				
	m/min	mm/rev	mm	wt %	T1	T2	T3	Avg	
1	150	0.07	0.90	0.30	0.43	0.45	0.42	0.44	30
2	165	0.03	0.70	0.60	0.45	0.39	0.41	0.42	26
3	150	0.07	0.90	0.90	0.38	0.38	0.37	0.37	23
4	165	0.07	0.70	0.60	0.48	0.45	0.55	0.48	31
5	180	0.07	0.90	0.90	0.61	0.62	0.62	0.62	24
6	180	0.03	0.90	0.90	0.50	0.53	0.52	0.53	25
7	180	0.03	0.50	0.90	0.42	0.40	0.38	0.39	23
8	180	0.07	0.50	0.90	0.45	0.34	0.46	0.46	24
9	165	0.05	0.90	0.60	0.69	0.73	0.73	0.71	30
10	165	0.05	0.70	0.60	0.53	0.39	0.69	0.64	28
11	180	0.05	0.70	0.60	0.52	0.51	0.63	0.58	26
12	180	0.07	0.50	0.30	0.55	0.44	0.55	0.48	28
13	165	0.05	0.70	0.60	0.63	0.61	0.62	0.62	25
14	165	0.05	0.50	0.60	0.50	0.50	0.63	0.57	24
15	150	0.05	0.70	0.60	0.36	0.40	0.36	0.37	28
16	180	0.03	0.90	0.30	0.73	0.75	0.70	0.70	26
17	150	0.07	0.50	0.90	0.31	0.35	0.36	0.36	27
18	180	0.03	0.50	0.30	0.36	0.35	0.37	0.43	28
19	165	0.05	0.70	0.60	0.57	0.65	0.57	0.61	25
20	165	0.05	0.70	0.30	0.79	0.76	0.74	0.79	29
21	165	0.05	0.70	0.60	0.56	0.93	0.69	0.62	28
22	165	0.05	0.70	0.60	0.77	0.47	0.48	0.63	29
23	150	0.07	0.50	0.30	0.30	0.34	0.34	0.32	32
24	180	0.07	0.90	0.30	0.76	0.66	0.71	0.74	27
25	150	0.03	0.90	0.30	0.46	0.47	0.44	0.41	35
26	150	0.03	0.90	0.90	0.23	0.23	0.29	0.29	25
27	150	0.03	0.50	0.90	0.49	0.49	0.50	0.30	26
28	150	0.03	0.50	0.30	0.23	0.29	0.22	0.29	30
29	165	0.05	0.50	0.30	0.73	0.76	0.74	0.70	38
30	165	0.05	0.70	0.90	1.04	0.95	0.96	0.73	26

#### 3.1.1 Analysis of variance (ANOVA)

##### 3.1.1.1 Analysis of variance for surface roughness

Table 6 presents the ANOVA statistical analysis of surface roughness, indicating a significant Model F-value (832.14) with only 0.01 % attributed to random noise, and p-values <0.0500, suggesting important terms in the model. Additionally, a lack-of-fit F value of 0.23 suggests a non-significant lack of fit compared to pure error, desirable for a well-fitting model, with a probability of 97.57 %. The corresponding regression equation developed of surface roughness is illustrated in Equation (4).

(4) $$\text{Ra}=-18.19894+0.219318V+42.19947f-1.87004d-1.12451\phi +0.010417V*f+0.012292V*d-0.002917V*\phi +0.468750f*d+1.56250f*\phi -0.493787d*\phi -0.000667V^{2}-437.67176f^{2}+0.322844d^{2}+1.47682\phi^{2}$$

**Table 6:** ANOVA for surface roughness

Source	Sum of Squares	df	Mean Square	F-value	p-value	
Model	0.6613	14	0.0472	832.14	<0.0001	significant
A-Cutting speed	0.0743	1	0.0743	1308.51	<0.0001	
B-Feed rate	0.0717	1	0.0717	1262.58	<0.0001	
C-Depth of cut	0.0000	1	0.0000	0.8636	0.3674	
D-Concentration	0.0318	1	0.0318	560.73	<0.0001	
AB	0.0002	1	0.0002	2.75	0.1179	
AC	0.0218	1	0.0218	383.25	<0.0001	
AD	0.0028	1	0.0028	48.55	<0.0001	
BC	0.0001	1	0.0001	0.9909	0.3353	
BD	0.0014	1	0.0014	24.77	0.0002	
CD	0.0146	1	0.0146	256.78	<0.0001	
A^2^	0.0646	1	0.0646	1138.26	<0.0001	
B^2^	0.0879	1	0.0879	1549.10	<0.0001	
C^2^	0.0005	1	0.0005	8.35	0.0112	
D^2^	0.0502	1	0.0502	884.50	<0.0001	
Residual	0.0009	15	0.0001			
Lack of Fit	0.0003	11	0.0000	0.2318	0.9757	not significant
Pure Error	0.0005	4	0.0001			
Cor Total	0.6622	29				

##### 3.1.1.2 Analysis of variance for tool tip temperature

This section presents statistics and tests of the tool tip temperature, with the smallest p-values indicating essential model terms. In Table 7, the ANOVA for tool tip temperature reveals a significant model F-value of 28.26, with only a 0.01 % chance of noise. If values exceed 0.1000, terms are considered insignificant, and model reduction may enhance effectiveness. A lack of fit F-value of 0.35 suggests insignificance, indicating a 92.58 % chance of random variation. The corresponding regression equation for tool tip temperature is illustrated in Equation (5).

(5) $$T=+58.23444+0.250173V+201.80231f-92.98914d-51.61288\phi +1.40000V*f+0.137917V*d+0.231667V*\phi -117.50000f*d-35.20833f*\phi +2.54213d*\phi -0.001913V^{2}-3413.71757f^{2}+51.86844d^{2}+5.77486\phi^{2}$$

**Table 7:** ANOVA for tool tip temperature

Source	Sum of Squares	df	Mean Square	F-value	p-value	
Model	158.31	14	11.31	28.26	<0.0001	significant
A-Cutting speed	2.39	1	2.39	5.97	0.0274	
B-Feed rate	4.71	1	4.71	11.77	0.0037	
C-Depth of cut	14.15	1	14.15	35.37	<0.0001	
D-Concentration	14.50	1	14.50	36.25	<0.0001	
AB	2.82	1	2.82	7.05	0.0180	
AC	2.74	1	2.74	6.85	0.0194	
AD	17.39	1	17.39	43.46	<0.0001	
BC	3.53	1	3.53	8.83	0.0095	
BD	0.7140	1	0.7140	1.78	0.2015	
CD	0.3864	1	0.3864	0.9657	0.3413	
A^2^	0.5317	1	0.5317	1.33	0.2670	
B^2^	5.35	1	5.35	13.37	0.0023	
C^2^	12.23	1	12.23	30.58	<0.0001	
D^2^	0.7678	1	0.7678	1.92	0.1862	
Residual	6.00	15	0.4001			
Lack of Fit	2.93	11	0.2666	0.3474	0.9258	not significant
Pure Error	3.07	4	0.7673			
Cor Total	164.31	29				

### 3.2 Discussion of the results

#### 3.2.1 Surface roughness interaction analysis

##### 3.2.1.1 The interaction effect of cutting speed on surface roughness

**Fig. 8:**
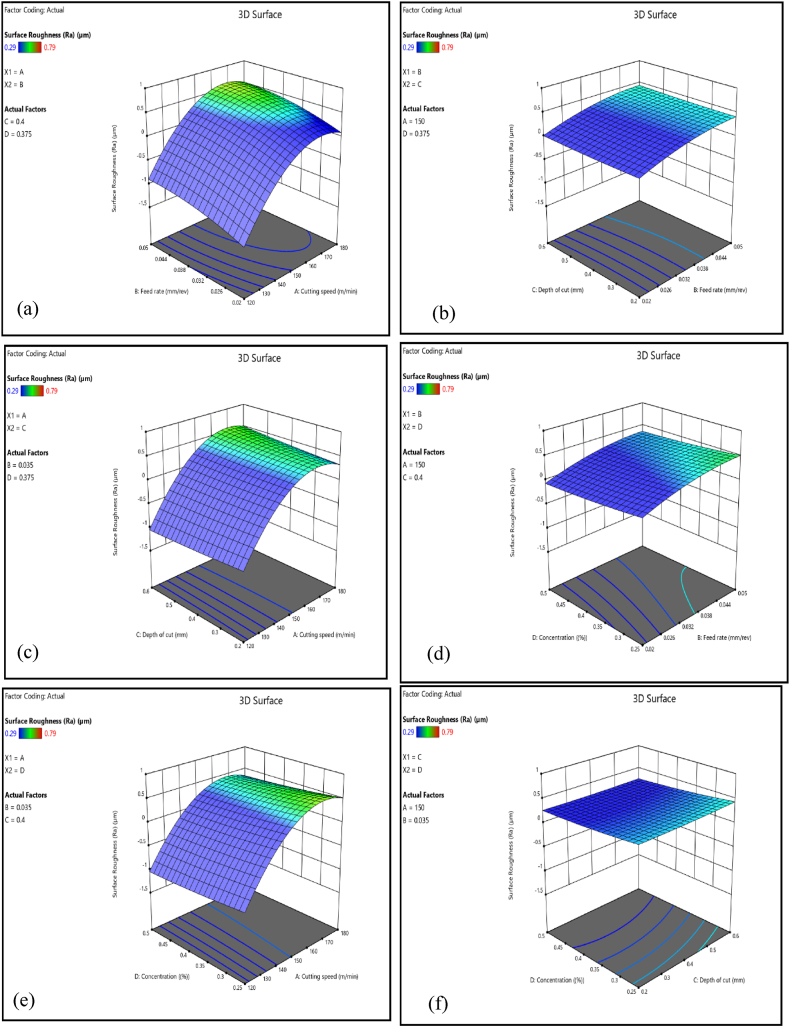
Cutting speed and feed rate interaction on surface roughness.

Surface roughness increases initially as cutting speed increases during the CNC turning of AISI D3 steel due to variables such as friction, heat generation, and tool wear. However, as the cutting speed approaches 160–170 m/min, maximum surface roughness is obtained, which thereafter begins to decline as shown in Fig. 8 (a), (b) and (c). This reduction is related to a balance in wear mechanisms, with higher speeds slowing tool wear and resulting in better surface quality. Existing literature supports this research by noting various factors that influence surface roughness during machining. Effective cooling techniques can greatly minimize tool wear, allowing for faster cutting speeds while maintaining surface quality [79,80]. Initially, faster cutting speeds generate more friction and heat, which accelerates tool wear and increases surface roughness [80]. However, wear mechanisms like abrasion and adhesion stabilize at optimal speeds, decreasing tool wear and enhancing surface quality [81]. Therefore, cutting speed had a significant impact on surface roughness, contributing to 11.45 % of the overall results, as shown in Table 6.

##### 3.2.1.2 The interaction effect of feed rate on surface roughness

In CNC turning of AISI D3 steel, increasing the feed rate generally results in higher surface roughness, as seen in Fig. 8 (a), (d), and (e). This increase in roughness is caused by more prominent scallop marks developing on the machined surface at higher feed rates. Additionally, feed rate has a major influence on chip flow and surface finish; greater feed rates frequently result in poorer surface quality because it leads to the formation of continuous, thick chips. Higher feed rates produce greater cutting forces, which directly contribute to tool wear [37,82]. The cutting force can be predicted based on characteristics such as tool shape and material behaviors, implying that optimizing feed rates can reduce tool wear. Higher feed rates can also cause vibrations and chatter, which not only degrade surface smoothness but also lead to potential tool damage [83]. Furthermore, greater feed rates contribute to more heat generation during the cutting operation. This excess heat may damage surface quality by inducing thermal deformation and affecting material properties at the surface [84]. As a result, while increased feed rates can increase production, they also pose major risks to tool longevity and machining quality. To achieve the optimal balance between efficiency and tool life, cutting conditions should be carefully optimized.

##### 3.2.1.3 The interaction effect of depth of cut on surface roughness

Deeper cuts in CNC turning can result in rougher surface finishes, as illustrated in Fig. 8 (b), (d) and (f), due to increasing cutting tool stresses. These stresses can produce uneven cutting and vibrations, reducing surface quality. Deeper cuts provide larger cutting forces, which can cause tool deflection, wear, and increased heat generation, distorting the workpiece and reducing surface quality [85]. This heat can cause thermal expansion and change material properties, resulting in defects [86]. Additionally, a higher depth of cut results in larger chips that are more difficult to evacuate efficiently [37]. If chips are recut between the tool and the workpiece, they can produce surface flaws and increase surface roughness. While deeper cuts can improve material removal rates and minimize machining time, they pose additional problems such as higher tool wear, heat generation, and potential surface defects. To optimize CNC turning processes, the depth of cut needs to be optimized with other machining variables. Thus, effective cooling methods are essential for enhancing tool performance and heat dissipation, extending tool life, and addressing these issues [87,88].

##### 3.2.1.4 The interaction effect of nanofluid concentration on surface roughness

An increase in hybrid nanofluids, such as those incorporating Al_2_O_3_ and graphene, can dramatically reduce surface roughness during machining processes, as illustrated in Fig. 8 (b), (d) and (f). These nanofluids behave as fillers, forming a thin lubricating layer between the tool and the workpiece, reducing direct contact, friction, and wear, and producing a smoother surface finish [89]. Thus, the combination of Al_2_O_3_ with graphene enhances the cutting fluid's lubrication and cooling capabilities. This hybrid enhancement helps to maintain lower temperatures while also reducing the thermal distortion of the workpiece, which is essential for obtaining a high-quality surface finish. The hybrid nanofluid's improved thermal conductivity contributes to more efficient heat dissipation and reduces surface roughness [90]. A hybrid nanofluid also improves tribological qualities, such as reduced friction and wear, through the formation of a protective tribo-film layer on the machined surface [91]. This layer prevents surface defects while improving overall surface quality. As a result, Al_2_O_3_/graphene hybrid nanofluids increase surface finishes by acting as effective fillers, increasing lubrication and cooling, and improving tribological properties, making them an important addition to modern machining processes. Moreover, well-dispersed Al_2_O_3_ and graphene nanoparticles in a fluid media can improve machining performance by lowering surface roughness and cutting forces [92].

#### 3.2.2 Tool tip temperature interaction analysis

##### 3.2.2.1 The interaction effect of cutting speed on tool tip temperature

**Fig. 9:**
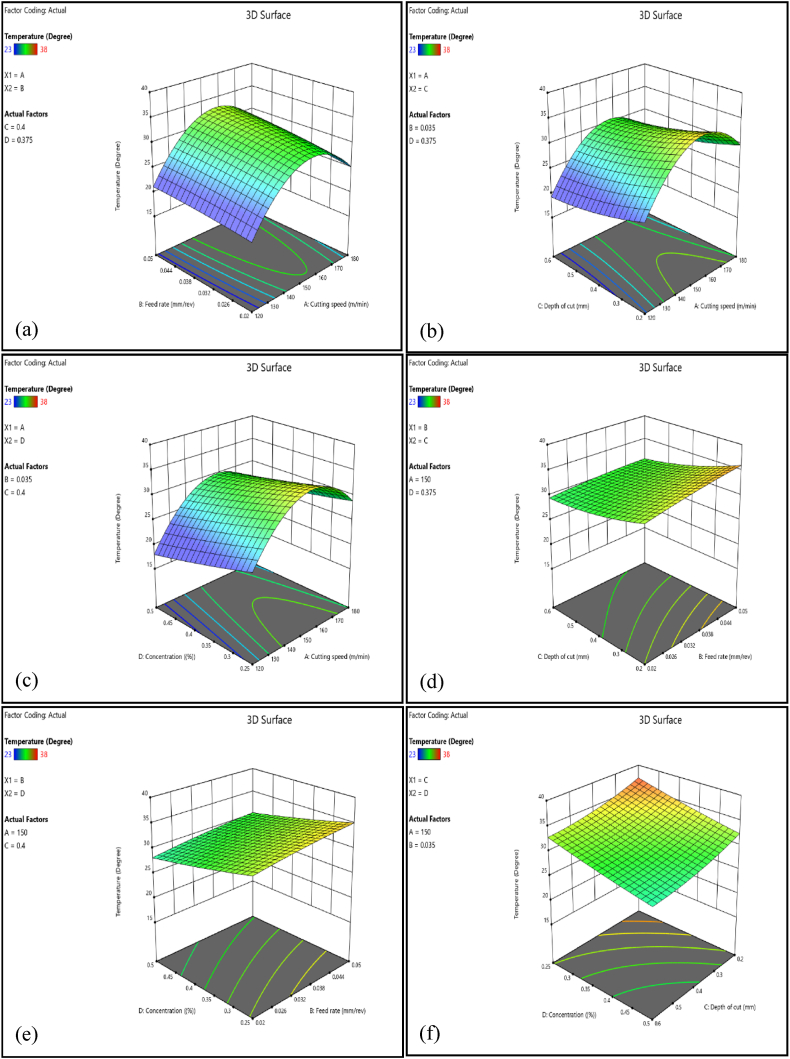
Interaction effect of cutting speed and depth of cut on the tooltip temperature.

Increasing the cutting speed in CNC turning increases friction and energy transfer between the workpiece and the cutting tool, resulting in higher temperatures near the tool tip [93]. This is mostly owing to the conversion of mechanical energy to heat, with friction at the tool-chip interface playing a significant role [94]. Typically, the maximum temperature is obtained at cutting speeds ranging from 150 to 160 m/min. Then, beyond this value, additional speed increases may not appreciably increase the temperature; in some cases, it may even fall slightly due to enhanced chip flow and cooling effects, as illustrated in Fig. 9 (a), (b), and (c). As the material is removed, chips carry away heat, contributing to temperature reduction [95]. However, when speeds exceed specific thresholds, a drop in cutting energy and effective strain is observed, which can contribute to lower temperatures [96]. Furthermore, modulated turning incorporates periodic cool-down intervals, which may significantly decrease both the maximum and average temperatures at the tooltip, increasing the tool's wear resistance [97].

##### 3.2.2.2 The interaction effect of feed rate on tool tip temperature

Increasing the feed rate in a CNC turning process can considerably increase the tool tip temperature, as illustrated by Fig. 9 (a), (d), and (e). This occurs because a faster feed rate increases the amount of material removed per unit time, resulting in increased cutting forces and heat at the tool-workpiece interaction [98,99]. Friction and distortion in the cutting zone allow mechanical energy to be converted into thermal energy, resulting in increased temperature. In addition as the feed rate increases, the tool engages with more material, resulting in increased friction, distortion, and consequently, greater temperature. Optimizing the feed rate, as well as other machining parameters, is crucial for efficiently managing tool tip temperature [98]. While a faster feed rate can increase productivity by minimizing machining time, it is essential to monitor the tool tip temperature to avoid excessive wear or damage [99]. As a result, while increasing the feed rate in CNC turning may increase tool tip temperature, proper optimization and monitoring can help minimize potential negative effects while ensuring efficient and effective machining processes.

##### 3.2.2.3 The interaction effect of depth of cut on tool tip temperature

The heat generated during cutting can be influenced by factors such as cutting speed, feed rate, depth of cut, and material characteristics, making it difficult to predict the intensity and distribution of heat during machining [100]. Increasing the depth of cut in machining operations has a considerable effect on tool temperature due to variations in heat dissipation and cutting forces. When the depth of cut varies from 0.4 to 0.5 mm, it can help reduce tool tip temperature by enhancing heat evacuation via greater chip formation, as shown in Fig. 9 (b), (d), and (f). This is due to a direct relationship between chip thickness and heat dissipation efficiency [101]. Larger chips remove more heat from the cutting zone, a concept confirmed by semi-analytical models that emphasize the need for efficient heat flux management [102]. Additionally, optimal coolant flow dynamics at the cutting edge can significantly enhance heat dissipation, particularly under high-pressure conditions [87,103]. However, increasing the depth of cut beyond 0.5 mm may result in increased tool tip temperatures due to more intense cutting conditions. This temperature increase could be due to an excessive depth of cut, which creates greater thermal loads [103]. As a result, precise control of cutting conditions needs to be done to minimize temperature distribution that could damage the tool and workpiece.

##### 3.2.2.4 The interaction effect of nanofluid concentration on tool tip temperature

Lower concentrations of hybrid nanofluids, which include Al_2_O_3_ and graphene, can result in higher temperatures at the tool-workpiece interface, as illustrated in Fig. 9 (c), (e) and (f). At lower concentrations, reduced nanoparticles in the fluid may limit its capacity to absorb and transfer heat effectively, resulting in greater temperatures during machining [104]. The addition of Al_2_O_3_ nanoparticles increases thermophysical parameters such as thermal conductivity, density, viscosity, and specific heat, all of which are necessary for efficient heat transfer and cooling in machining processes [105]. Graphene-based nanofluid, in particular, is known for its high thermal conductivity, which is critical for increasing heat transfer efficiency [106]. According to Kumar et al. [107] a hybrid nanofluid comprising graphene oxide and MXene increased electrical conductivity by 20.5 % and thermal conductivity by 6.81 % at 45 °C. Thus the hybrid of Al_2_O_3_ and graphene nanoparticles greatly improves the fluid's thermophysical characteristics, resulting in lower cutting temperatures and reduced surface roughness [108]. Tiwari et al. [109] found that using Al₂O₃ nanofluid reduces machining temperatures in steel by 55 % compared to dry cutting, 38 % compared to flood cooling, and 20 % compared to coconut oil with minimal lubrication. Sharma et al. [110] predicted that the optimal temperature range for cryogenic turning of AISI D3 steel was 24.44 °C–25.54 °C.

However, the maximum temperature recorded during the experiments was 42 °C. While cryogenic cooling is effective at minimizing temperatures, the use of Al_2_O_3_ and graphene nanoparticles in nanofluids provides additional benefits beyond it. These advantages include not only lower temperatures but also improved tribological properties, which cryogenic methods alone may not address as effectively. As a result, the maximum temperature reached in this study with these nanofluids was reduced to 38 °C, demonstrating its superior performance in controlling temperature during machining operations.

#### 3.2.3 Artificial neural network (ANN)

**Fig. 10:**
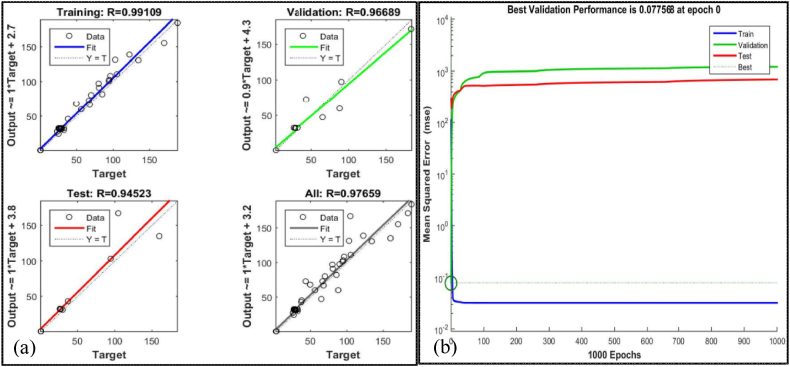
ANN results and regression diagrams.

**Fig. 11:**
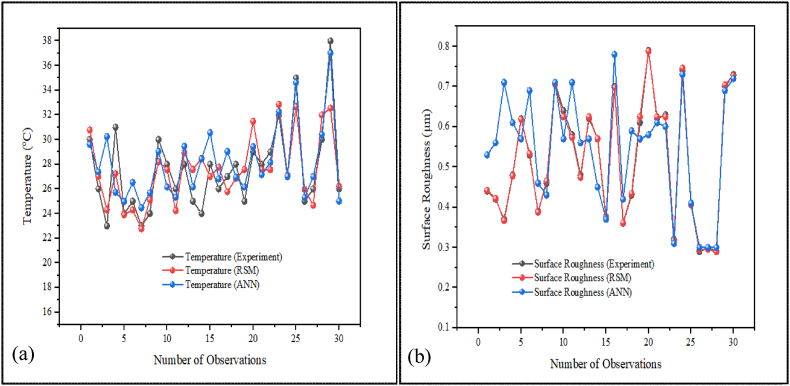
Comparative results of the experiment, RSM, and ANN for (a) tool tip temperature and (b) surface roughness.

The incorporation of ANN in CNC turning processes significantly increases productivity, accuracy, and flexibility in modern manufacturing. ANNs improve optimization and innovation by incorporating machine learning and data analysis, making them indispensable for advanced machining operations. The ANN can effectively predict optimal measurement times in CNC machining, reducing downtime and improving operational efficiency [111]. It is also more effective in predicting surface roughness (Ra) than traditional methods, with a lower error margin [112,113]. The use of intelligent CNC technology, along with big data and artificial intelligence, is essential for long-term development in the machinery manufacturing sector. This approach improves product accuracy, reduces production cycles, and enhances overall efficiency [114]. Based on Table 8 summarizes the ANN predicted results while Fig. 10 (a) shows training data, predicted outcomes, and performance verification of simulation results, with the greatest results achieved by the ANN after 1000 iterations. ANN demonstrates adaptability and accuracy by consistently achieving high R-squared values (close to one) and minimum mean square errors (close to zero) across multiple datasets [30,115]. These metrics show an excellent fit between the model and the data, confirming the efficiency of ANN in achieving optimal results. The average R-squared values greater than 0.97 indicate highly accurate fitting across all datasets, affirming the model's adaptability. Quality assessment using MATLAB 2022 R2 regression graphs confirmed the ANN's prediction accuracy, as shown in Fig. 10 (b), which includes a regression plot and an acceptable mean square error. The comparison between the experimental results, the RSM, and ANN outputs is depicted in Fig. 11 (a) and (b). The outcomes indicate that the experimental results confirmed that no variation from the predicted results by RSM and ANN. Consequently, the consistency between the experimental and anticipated results from RSM and ANN demonstrates the accuracy of the experimental data and the reliability of these predictive models.

**Table 8:** ANN predicted results

Run	v	f	d	ϕ	Ra (ANN Predicted)	T (ANN Predicted)
	m/min	mm/rev	mm	wt %	μm	°C
1	150	0.07	0.90	0.30	0.53	29.61
2	165	0.03	0.70	0.60	0.56	27.39
3	150	0.07	0.90	0.90	0.71	30.24
4	165	0.07	0.70	0.60	0.61	25.72
5	180	0.07	0.90	0.90	0.57	24.97
6	180	0.03	0.90	0.90	0.69	26.53
7	180	0.03	0.50	0.90	0.46	24.47
8	180	0.07	0.50	0.90	0.43	25.68
9	165	0.05	0.90	0.60	0.71	29.04
10	165	0.05	0.70	0.60	0.57	26.15
11	180	0.05	0.70	0.60	0.71	25.33
12	180	0.07	0.50	0.30	0.56	29.46
13	165	0.05	0.70	0.60	0.57	26.15
14	165	0.05	0.50	0.60	0.45	28.44
15	150	0.05	0.70	0.60	0.37	30.56
16	180	0.03	0.90	0.30	0.78	26.81
17	150	0.07	0.50	0.90	0.42	29.05
18	180	0.03	0.50	0.30	0.59	26.95
19	165	0.05	0.70	0.60	0.57	26.15
20	165	0.05	0.70	0.30	0.58	29.43
21	165	0.05	0.70	0.60	0.61	27.15
22	165	0.05	0.70	0.60	0.60	28.15
23	150	0.07	0.50	0.30	0.31	32.27
24	180	0.07	0.90	0.30	0.73	27.03
25	150	0.03	0.90	0.30	0.41	34.63
26	150	0.03	0.90	0.90	0.30	25.31
27	150	0.03	0.50	0.90	0.30	26.99
28	150	0.03	0.50	0.30	0.30	30.41
29	165	0.05	0.50	0.30	0.69	37.03
30	165	0.05	0.70	0.90	0.72	25.02

#### 3.2.4 Validation of the model

**Fig. 12:**
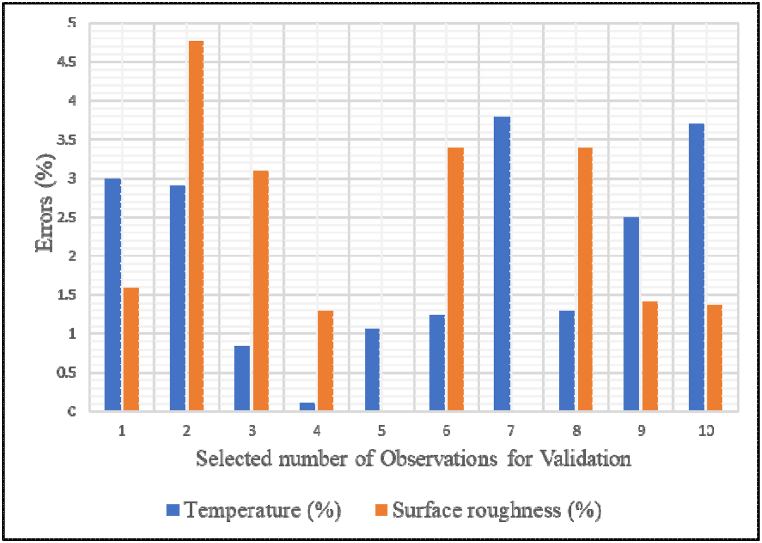
Errors comparison in the validation.

The last 10 values (runs 21–30) of results were selected from the experimental and ANN results to validate the observed results [30]. Table 9 indicates predicted average errors, both temperature and surface roughness average error percentages are within ±2 %; which defines the validity and accuracy of the work. Supporting this, Šarić et al. [116] reported that ANN models achieved error rates as low as 2.26 % for surface roughness, while Manjunatha [112] found errors below 3.45 %. The corresponding comparison of the errors observed from temperature and surface roughness is shown in Fig. 12.

**Table 9:** The experimental and ANN results in errors comparison

Run	v	f	d	ϕ	Responses				Error (%)	
					Ra (Experiment)	T (Experiment)	Ra (ANN)	T (ANN)	Ra	T
	m/min	mm/rev	mm	wt %	μm	°C	μm	°C	%	%
21	165	0.05	0.70	0.60	0.62	28	0.61	27.15	1.6	3.0
22	165	0.05	0.70	0.60	0.63	29	0.60	28.15	4.76	2.9
23	150	0.07	0.50	0.30	0.32	32	0.31	32.27	3.1	0.84
24	180	0.07	0.90	0.30	0.74	27	0.73	27.03	1.3	0.11
25	150	0.03	0.90	0.30	0.41	35	0.41	34.63	0.0	1.05
26	150	0.03	0.90	0.90	0.29	25	0.30	25.31	3.4	1.24
27	150	0.03	0.50	0.90	0.3	26	0.3	26.99	0.0	3.8
28	150	0.03	0.50	0.30	0.29	30	0.3	30.41	3.4	1.3
29	165	0.05	0.50	0.30	0.7	38	0.69	37.03	1.42	2.5
30	165	0.05	0.70	0.90	0.73	26	0.72	25.02	1.36	3.7
% of error									2.0	2.0

#### 3.2.5 Genetic algorithm (GA)

**Fig. 13:**
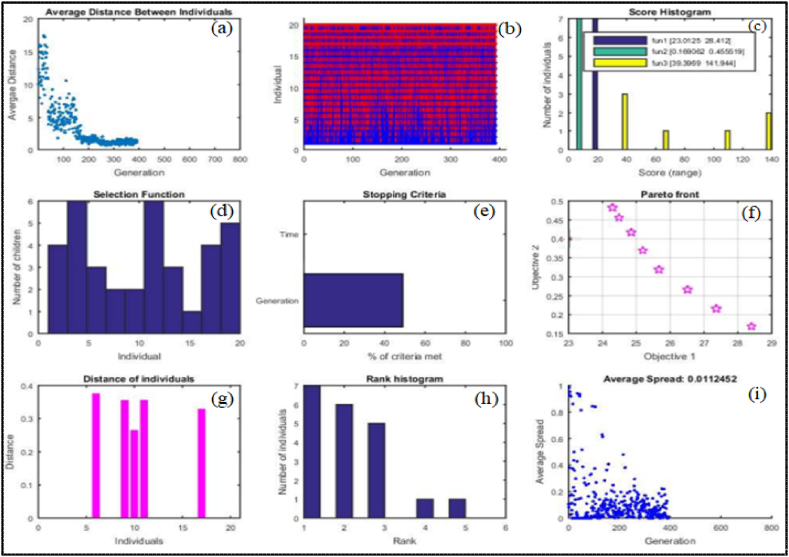
GA results for the optimal solution: (a) average distance of individuals per generation, (b) cross-selection results, (c) final objective function values, (d) progeny count per individual, (e) stop condition, (f) Pareto front, (g) final generation distances, (h) histogram of individual fitness in the final population, (i) average algorithm spread across runs.

Implementing GA in CNC turning increases machining productivity, accuracy, and flexibility by optimizing cutting parameters and reducing errors [117]. A genetic algorithm enhances the selection of machining parameters, resulting in greater processing performance and more accurate predictions than traditional methods [118]. Table 10 illustrates the surface roughness from the selected parameters integrated with the concentration of nanoparticles. The predicted GA results for the optimal solution are presented in Fig. 13, panels (a)–(i). The optimum solution to the GA fitness functions, attained after 400 generations was an average cutting speed of 150 m/min, feed rate of 0.05 mm/rev, depth of cut of 0.6 mm and nanoparticle concentration of 0.8 %. Using these settings, the optimal temperature ranges from 23.01 °C to 28.41 °C, with a desired surface roughness of 0.16–0.45 μm.

**Table 10:** Optimal predicted parameters (GA)

Run	v	f	d	ϕ	Ra (GA predicted)	T (GA predicted)
	m/min	mm/rev	mm	wt %	μm	°C
1	152.73	0.06	0.75	0.86	0.39	23.02
2	150.32	0.03	0.53	0.60	0.16	28.40
3	150.32	0.03	0.53	0.60	0.16	28.40
4	152.68	0.05	0.75	0.86	0.45	24.84
5	150.50	0.05	0.55	0.61	0.34	28.48
6	152.74	0.06	0.75	0.87	0.39	23.01
7	150.32	0.03	0.53	0.60	0.16	28.41
8	152.74	0.06	0.75	0.87	0.39	23.01
9	150.87	0.03	0.75	0.86	0.36	25.18
10	150.58	0.04	0.57	0.84	0.40	26.58
11	150.69	0.03	0.69	0.73	0.29	25.97
12	150.32	0.03	0.53	0.60	0.17	28.41
13	150.32	0.03	0.53	0.60	0.16	28.40
14	152.74	0.06	0.75	0.87	0.39	23.01
15	152.03	0.05	0.75	0.86	0.45	25.23
16	152.74	0.06	0.75	0.86	0.39	23.02
17	151.45	0.06	0.58	0.69	0.34	26.80
18	151.83	0.06	0.65	0.86	0.45	24.48
19	150.32	0.03	0.53	0.60	0.16	28.41
20	152.74	0.06	0.75	0.87	0.39	23.01

## 4 Conclusion

This study effectively combined RSM with numerous machine learning approaches, including ANN and GA, to improve cutting parameters and reduce surface roughness in machining operations. The study highlights the importance of cutting speed, feed rate, depth of cut, and the concentration of hybrid (Al_2_O_3_/graphene) nanofluids on surface roughness and tool tip temperature. Therefore, the most important results of this study are summarized below.

• Cutting speed has a considerable influence on surface roughness, and nanofluid concentration plays an essential role in sustaining tool tip temperature.
• Higher concentrations of hybrid (Al_2_O_3_/graphene) nanofluid help achieve smoother surfaces by minimizing friction, reducing wear, improving heat conductivity, establishing a protective layer, and increasing viscosity. This reduces surface roughness and improves heat dissipation, hence lowering frictional heat. Lower concentrations, on the other hand, cause higher temperatures due to enhanced heat transmission and reduced heat dissipation.
• The experimental results closely match the predictions of the RSM and ANN models, exhibiting consistency and confirming the experimental data's accuracy. The predicted average errors for temperature and surface roughness are within ±2 %, indicating the prediction models' validity.
• The GA determined that the optimal input variables for the process were a cutting speed of 150 m/min, a feed rate of 0.05 mm/rev, a cut depth of 0.6 mm, and a nanoparticle concentration of 0.8 %. The temperature ranges from 23.01 °C to 28.41 °C, achieving a desired surface roughness of 0.16–0.45 μm.

This work demonstrates that hybrid nanofluids and machine learning algorithms can efficiently optimize CNC turning operations by managing temperature and surface roughness. Future study needs to investigate how these approaches work with different materials, machining parameters, and nanofluids.

## CRediT authorship contribution statement

**Leta Daba Gemechu:** Writing – original draft, Resources, Data curation, Conceptualization. **Dame Alemayehu Efa:** Writing – review & editing, Visualization, Validation, Supervision, Software, Methodology, Investigation, Formal analysis, Conceptualization, Data curation, Project administration, Resources, Writing – original draft. **Robsan Abebe:** Writing – review & editing, Project administration, Investigation, Funding acquisition, Data curation, Formal analysis.

## Data and code availability statement

The data that has been used is confidential.

## Funding

No funding was available for this research work.

## Declaration of competing interest

The authors declare that they have no known competing financial interests or personal relationships that could have appeared to influence the work reported in this paper.
